# Just Missing the Mark: Discharging High-risk Atrial Fibrillation / Flutter without Thromboprophylaxis

**DOI:** 10.5811/westjem.2018.2.37337

**Published:** 2018-02-26

**Authors:** Linda B. Thompson, Michael C. Kurz

**Affiliations:** University of Alabama School of Medicine, Department of Emergency Medicine, Birmingham, Alabama

Atrial fibrillation and flutter (AF) is a pervasive disease affecting 6.1 million people in the United States.^1^ Each year it is responsible for more than 750,000 hospitalizations and 130,000 deaths.^2^,^3^ In contrast to overall declining death rates for cardiovascular disease,^4^ AF as the “primary or contributing cause of death has been rising for more than two decades.”^3^ The annual economic burden of AF is six billion dollars; medical costs per AF patient are about $8,707 higher than for non-AF individuals.^3^

Thrombotic embolism of the cerebral circulation, or stroke, is the principal risk of AF and ranges from less than 2% to greater than 10% annually.^5^–^8^ AF is the cause of 100,000–125,000 embolic strokes each year, of which 20% are fatal.^9^ Anticoagulation to prevent these embolic events is standard of care unless contraindicated.^9^ However, it is not without risk, as even minor trauma can cause substantial and potentially life-threatening bleeding. Given that AF is the most common arrhythmia among the elderly,^1^,^2^,^3^ balancing these competing risks is challenging.

Anticoagulation for AF is most commonly accomplished with a vitamin K antagonist, warfarin. However, its use requires patient education, medication compliance, dietary consistency, and close monitoring. CHA_2_DS_2_-VASc, ATRIA, HAS-BLED, ORBIT, and HEMORR_2_HAGES are just some of the decision-support tools available to objectively weigh the risk of stroke and life-threatening bleeding from therpy.^10^–^15^ Newer, novel oral anticoagulant agents (NOAC) provide a benefit/risk profile that may surpass warfarin, especially when considering initiation in the emergency department (ED). 16–18

In this issue of *WestJEM*, Smith and colleagues present a prospective observational evaluation of anticoagulation prescribing practices in non-valvular AF. Patients presenting to one of seven Northern California EDs with AF at high risk for stroke were eligible unless admitted, not part of Kaiser Permanente of Northern California (KPNC), or already prescribed anticoagulation. During the 14-month study there were no departmental policies governing the initiation of anticoagulation in AF patients.

The authors report 27.2% of the 312 at high risk for stroke received a new anticoagulant at ED discharge, and only 40% were prescribed oral anticoagulation within 30 days of the index ED visit. Anticoagulation was more likely to be initiated in the ED if the patient was younger (age < 80), had persistent AF at discharge, or when cardiology was consulted during the index visit. Furthermore, only 60.3% of patients were given patient education material on AF in their discharge instructions.^19^

Critics of Smith et al. will take issue with their inclusion criteria that required participation in KPNC. By definition, all members of KPNC are insured; they also have guaranteed access to timely primary care follow-up and are of higher socioeconomic means than the general population.^20^ Many of the factors that contribute to successful anticoagulation therapy – diet stability, monitoring of renal function, education and intervention of modifiable risk factors, smoking cessation, and fall risk – can all be assessed by a primary care physician and addressed with shared decision-making ensured in the KPNC system.^21^

While these limitations are acknowledged by the authors and narrow the generalizability of these findings, Smith and colleagues demonstrate the challenges of addressing ongoing chronic disease in the ED and highlight the complex decision-making required. AF patients without insurance in the U.S. lack reliable access to primary care, and emergency physicians (EPs) likely under-prescribe anticoagulation therapy due to an abundance of caution. EPs are poorly equipped to determine the burden of AF (i.e., is this isolated AF or recurrent and how often is the patient in it) or the origin of the arrhythmia (i.e., is it valvular?). Lacking the objective data to quantify these thromboembolic risk factors of AF, EPs are reluctant to initiate thromboprophylaxis, despite its known benefits, in light of the well-demonstrated risk for life-threatening bleeding.^22^

However, the risk is largely misperceived. Recent findings from the Spanish EMERG-AF trial demonstrate that initiating this therapy in the ED is at least as safe as in other settings (i.e., in the outpatient clinic or during an inpatient stay) and has clear mortality benefit at one year. Furthermore, that benefit (i.e., stroke prevention) does not come at the expense of reduced effectiveness (i.e., more episodes of major bleeding) over the course of one-year follow-up.^23^

In addition to highlighting the challenges of prescribing anticoagulation in the ED setting, Smith et al. also illustrate the opportunity for EPs to prevent future strokes in the setting of known AF. This opportunity is likely larger than reported considering the limitations of this investigation (i.e., enrollment predicated upon KPNC participation). Thankfully, there are clear guidelines to assist EPs based upon validated methods of risk-stratification.^24^,^25^ Furthermore, of those patients receiving anticoagulation therapy in the first 30 days, more than half were initiated in the ED. While these subjects likely represent the least complex decision-making, these results also suggest some prescribing inertia; anticoagulation was continued by the primary care physician because it has already been initiated in the ED.

Despite these limitations, Smith and colleagues demonstrate an immense target for EPs to improve stroke risk for at least 60% of AF patients discharged from the ED. Coupled with other evidence demonstrating that such practice is efficacious, safe, and cost effective, Smith makes a compelling case that thromboprophylaxis should be initiated in all but the most complex AF patients who will likely be admitted. EDs should develop policies to assure that AF patients can receive anticoagulation therapy on discharge. These local policies could include decision pathways that rely on guidelines, decision-support tools, and account for insurance status. As EPs, we should embrace the responsibility to provide thromboprophylaxis regardless of the likelihood of primary care follow-up. To defer that decision ignores the role emergency medicine plays in providing for the public health in the U.S., and frankly misses the mark.
